# Health-Related Quality of Life in Pediatric Inflammatory Bowel Disease Patients: A Narrative Review

**DOI:** 10.7759/cureus.29282

**Published:** 2022-09-18

**Authors:** Saad Ahmed, Sadia Alam, Mohammed Alsabri

**Affiliations:** 1 Medicine, SUNY Downstate College of Medicine, Brooklyn, USA; 2 Emergency Medicine, Al Thawra Modern General Hospital, Sana'a, YEM

**Keywords:** heath related quality of life, pediatrics gastroenterology, crohn’s disease (cd), ulcerative colitis, inflammatory bowel disease

## Abstract

Inflammatory bowel disease (IBD) is a chronic autoimmune condition that can have a wide range of symptoms among pediatric patients. Although clinical symptoms like hematochezia, diarrhea, and abdominal pain are commonly addressed, health-related quality of life (HRQOL) is often overlooked in patients with IBD and pediatric patients with chronic disease in general. Examining HRQOL can help improve patient outcomes, but it has been studied sparingly. In this review, we aim to compare HRQOL between pediatric patients suffering from IBD and healthy children, as well as those suffering from other illnesses. We searched through peer-reviewed primary literature related to IBD and HRQOL and selected 10 articles from the PubMed database to be reviewed. Our inclusion criteria included articles published after the year 2000 in English, primary studies, and those that corresponded to the aim of this review. Case reports and secondary and tertiary articles were excluded from our review. We found that patients with IBD reported worse HRQOL in terms of overall health and in various subdomains, including physical health and fatigue, compared to their healthy counterparts. However, children with IBD demonstrated a comparable HRQOL with children suffering from functional abdominal pain (FAP) and obesity. Additionally, children with IBD displayed a greater HRQOL than pediatric patients with gastroesophageal reflux disease (GERD) and chronic constipation. In addressing the aim of this review, we found that children with IBD had a lower HRQOL when compared to healthy children, but a comparable or greater HRQOL than other sick children. Some factors associated with a reduced HRQOL include disease activity, age, fatigue, gender, psychological variables, and associated symptoms. Going forward, HRQOL should be considered by practitioners when caring for pediatric IBD patients in a clinical setting as it can help improve patient care. More studies need to be conducted to further explore HRQOL in pediatric patients. This can help implement early psychosocial interventions in children to reduce the disease burden.

## Introduction and background

Inflammatory bowel disease (IBD), including both ulcerative colitis (UC) and Crohn’s disease, is a chronic immune-mediated condition that can have debilitating symptoms and sequelae. The prevalence of IBD is on the rise in the United States as 77 per 100,000 American children are diagnosed with this illness [1]. It is estimated that at least 25% of IBD cases are found in the pediatric population [2]. Patients suffering from IBD experience symptoms of abdominal pain, diarrhea, hematochezia, weight loss, and fatigue [3,4]. Moreover, symptoms of pain are often present even in the absence of flares and while the illness is in remission due to changes in sensory pathways, pain processing, and neural connections [5]. By the same token, a significant portion of these patients suffers from anxiety and/or depression [6]. They are also more susceptible to developing psychiatric illnesses, such as bipolar disorder and schizophrenia, thereby worsening the burden of IBD [7]. Although there has been an increase in IBD cases, not much attention has been directed toward examining the quality of life (QoL) in these patients. QoL is a useful indicator in patient care because its improvement can lead to better outcomes.

QOL, as defined by the World Health Organization, is “an individual's perception of their position in life in the context of the culture and value systems in which they live and in relation to their goals, expectations, standards and concerns” [8]. Health-related quality of life (HRQOL) focuses more specifically on the physical, mental, and emotional aspects of one’s life that can affect and be affected by health status directly [8]. Some factors encompassed by HRQOL are symptoms of disease, treatment side effects, treatment satisfaction, life satisfaction, and emotional well-being. It is vital to examine HRQOL for a multitude of reasons. It can encourage a biopsychosocial approach to patient treatment and force us to examine the patient beyond the illness. It can serve as a marker of patient progress and improvement and help achieve patient comfort and wellness. Considering patient HRQOL can also enhance protective factors and minimize risk factors to improve the clinical course and prevent sequelae such as depression and anxiety. All of this is especially important in chronic illnesses, such as IBD, where clinical outcomes can vary greatly and symptoms can be persistent and severe. Similarly, studying HRQOL in pediatric patients should be emphasized since these patients are young and have much of their lives ahead of them. Pediatric patients also present with more extensive symptoms than adults [9]. Thus, managing IBD in pediatric patients should include an evaluation of HRQOL [10].

There are several reasons why incorporating HRQOL in patient care has been so lacking. For instance, HRQOL is often viewed as a subjective measure and is deemed less valuable than objective lab results. Similarly, HRQOL can vary between patients and thus have little clinical significance. Few efforts have also been made to quantify HRQOL, which can raise the question of how to properly examine HRQOL. Practicing medicine is also based on convention and protocol. In other words, if one aspect of patient care has been rarely done, it ceases to be part of the standard of care, such as incorporating HRQOL. However, despite these hesitancies, HRQOL should still be included in patient care. Furthermore, steps have been taken to improve the implementation and efficacy of HRQOL, such as through the use of surveys. These surveys include the Pediatric Quality of Life (PedsQL) questionnaire, IMPACT-III questionnaire, Children’s Depression Inventory, and Netherlands Organization for Applied Scientific Research Academic Medical Center Children's Quality of Life (TACQOL) questionnaire. These surveys are advantageous in examining HRQOL because they are validated, concise, standardized, inclusive, and thorough. They can be applied among children from different age groups and explore physical, social, and emotional functioning [11]. It is also beneficial to use a variety of surveys as they allow for a comprehensive review of HRQOL in these patients. The objective of this review is to examine HRQOL in pediatric patients suffering from IBD by comparing (1) HRQOL between pediatric IBD patients and healthy children and (2) HRQOL between pediatric IBD patients and other chronically ill children.

This article was previously posted to the ResearchSquare preprint server on April 14, 2022. It is not pending publication elsewhere.

## Review

Methods

**Figure 1:**
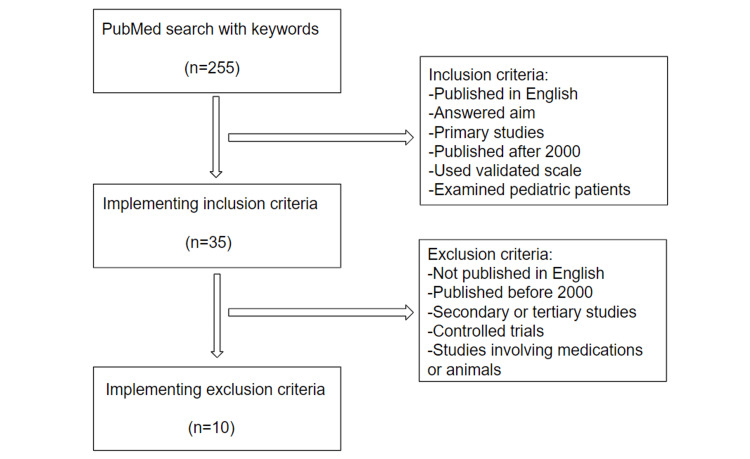
Flowchart detailing inclusion and exclusion criteria

Our search strategy included entering MeSH terms and keywords into the PubMed database to retrieve relevant articles for our review. The keywords used were (((((IBD) OR Crohn's) OR Ulcerative Colitis) AND Quality of life) OR Health-related quality of life) AND pediatrics. This generated a total of 255 articles. Then, the articles were screened to determine if they met the inclusion and exclusion criteria. The inclusion criteria involved primary studies analyzing HRQOL in pediatric patients with IBD and its subtypes in comparison to either healthy patients or other ill patients. Articles published in English after 2000 were included, along with studies that were case-controlled, cross-sectional studies, and those that measured HRQOL with a validated scale. The exclusion criteria included secondary or tertiary articles and control trials involving medications and animals. Case series and case reports were also excluded. Articles published before 2000, those not in English, and those examining adult populations were excluded. Articles studying psychological variables were also not included. Papers were also screened to determine if they offered information that would help answer the questions proposed by this review. A flowchart of our inclusion and exclusion criteria is presented in Figure 1.

Ultimately, 10 studies were selected for our review. Table 1 summarizes the extracted data from the 10 articles, which include the author list, publication year, study design, study setting, participant characteristics, sample size, type of QOL survey, and results.

**Table 1:** Reviewed articles comparing HRQOL in IBD, healthy, and other sick children HRQOL: health-related quality of life; IBD: inflammatory bowel disease; IGF-1: insulin-like growth factor 1; GERD: gastroesophageal reflux disease; UC: ulcerative colitis

Paper number	Title	Authors	Year	Country	Type of study	Objective	Patient groups	Questionnaire/survey	Overall outcome
1	Health-Related Quality of Life in Overweight/Obese Children Compared With Children With Inflammatory Bowel Disease [8]	Faus et al.	2014	USA	Cross-sectional study	To compare HRQOL in pediatric IBD patients with children who are obese/overweight	60 patients diagnosed with IBD and 60 patients who are overweight/obese with a BMI >85th percentile, all between the ages of 5 and 19 years	PedsQL 4.0	Children with IBD have HRQOL scores comparable to children who are obese or overweight
2	Quality of Life in Children and Adolescents With Inflammatory Bowel Disease: Impact and Predictive Factors [10]	Silva et al.	2020	Brazil	Cross-sectional, analytical case series	To determine the QoL of children and adolescents with known IBD and evaluate the factors that influence this QoL	35 children and adolescents between the ages of 3 and 18 years diagnosed with IBD were evaluated at a tertiary pediatric gastroenterology service in Brasília and compared to 62 healthy controls	PedsQL 4.0	IBD negatively affects the QoL of children and adolescents based on its impact on the physical, emotional, social, and psychosocial statuses of these patients
3	Measurement Properties of the UK-English Version of the Pediatric Quality of Life Inventory™ 4.0 (PedsQL™) Generic Core Scales [11]	Upton et al.	2005	UK	Cross-sectional study	To develop a UK-English version of the PedsQL and assess its validity by administering it in children with chronic conditions and healthy children and compare results to those of the validated PedsQL	76 pediatric patients with IBD, 1034 healthy controls, 99 pediatric patients with asthma, 124 children with diabetes, and 66 children with cancer, all within the age range of 5-18 years	PedsQL	Children with IBD showed lower PedsQL scores than healthy children on all scales except social functioning, where there was no statistical difference
4	Fatigue and Health-Related Quality of Life in Pediatric Inflammatory Bowel Disease [12]	Marcus et al.	2009	USA	Cross-sectional study	To evaluate the level of fatigue and assess HRQOL in children with IBD compared to healthy controls	70 children with IBD and 157 healthy children between 10-17 years of age	PedsQL Multidimensional Fatigue Scale, PedsQL 4.0	In children with primarily inactive IBD, fatigue was significantly higher and HRQOL was significantly lower than in healthy controls
5	Quality of life in Paediatric Inflammatory Bowel Disease Measured by a Generic and a Disease-Specific Questionnaire [13]	Loonen et al.	2002	Netherlands	Cross-sectional study	To assess HRQOL in children and adolescents with IBD by using both a generic and a disease-specific measurement tool	83 children between ages 8-18 with IBD from large secondary and tertiary centers in the Netherlands were compared to 1810 healthy controls	TACQOL for general QoL IMPACT-II for disease-specific QoL	Adolescents with IBD have a severely affected HRQOL. High occurrence of negative emotions places patients at risk for depressive and behavioral disorders
6	Low Insulin-like Growth Factor-1 Influences Fatigue and Quality of Life in Children With Inflammatory Bowel Disease [14]	Lucia Casadonte et al.	2018	USA	Prospective cohort study	To study the association between fatigue, IGF-1, and inflammatory cytokines in pediatric patients with IBD and compare QoL between pediatric IBD patients and healthy children	67 IBD patients (age 10-16) compared with 157 healthy patients (age 10-17) from a pediatric IBD subspecialty clinic in Chicago between September 2014 and February 2016	PedsQL, PedsQL Fatigue Scale, IMPACT-III, Children's Depression Inventory, Crohn's Disease Activity Index, UC Activity Index, and Physician Global Assessment	Children suffering from IBD experience statistically significantly more fatigue and worse HRQOL than healthy children
7	Reduced Quality of Life in Children With Gastro-Oesophageal Reflux Disease [15]	Marlais et al.	2010	UK	Cross-sectional study	To obtain and compare the self-reported QoL assessments in pediatric patients suffering from GERD and compare it with healthy children and pediatric patients suffering from IBD and chronic constipation	184 children including 40 with GERD, 44 with constipation, 59 with IBD, and 41 healthy children aged 5-18 from a tertiary pediatric GI clinic between February and May 2009	PedsQL	Children with IBD have a statistically significantly greater QoL than children with GERD
8	Quality of Life for Children With Functional Abdominal Pain: A Comparison Study of Patients’ and Parents’ Perceptions [16]	Youssef et al.	2005	USA	Cross-sectional study	To evaluate pediatric patients and their parents' perceptions regarding the QoL in children suffering from functional abdominal pain	65 pediatric patients with IBD vs. 65 pediatric patients with functional abdominal pain vs. 46 healthy children vs. 56 with GERD visiting a pediatric referral center	PedsQL	Children with IBD reported similar QoL scores as those with GERD, FAP, and healthy children
9	Chronic Childhood Constipation Is Associated With Impaired Quality of Life: A Case-Controlled Study [17]	Youssef et al.	2005	USA	Case-controlled study	To examine the impact of chronic constipation on the QoL in pediatric patients and compare it with healthy controls or patients with other chronic conditions	42 pediatric patients suffering from IBD, 80 children suffering from constipation, 56 children with GERD, and 46 healthy children aged 5-18 years visiting a tertiary pediatric care center	PedsQL	Pediatric patients with IBD demonstrate a statistically significantly higher QoL when compared with children suffering from chronic constipation
10	Disease Impact on the Quality of Life of Children With Inflammatory Bowel Disease [18]	Chouliaras et al.	2017	Greece	Cross-sectional study	To evaluate the impact of disease and factors influencing the QoL in children with IBD	99 IBD patients with a mean age of ~13 years and consisting of 37 patients with UC and 62 patients with Crohn's disease	IMPACT-III	Disease activity is the primary correlate of decreased QOL in children with IBD

Results

Based on the inclusion criteria, we selected 10 primary articles for our review as described above. From these articles, the extracted data included the author list, publication year, study design, study setting, participant characteristics, sample size, type of QOL survey, and results. The selected articles compared children diagnosed with IBD to healthy controls or other ill children by using questionnaires on disease impact and severity, including the PedsQL 4.0 and the IMPACT-III surveys. The PedsQL survey was designed to measure HRQOL in adolescents and children with both chronic and acute health conditions. It considers physical, emotional, social, and school functioning as well as physical health and psychosocial health. The higher the score, the better the HRQOL in the child/adolescent. The IMPACT-III questionnaire, similarly, is an HRQOL measure. However, it is specific for pediatric patients with diagnosed IBD. Both questionnaires have been established as valid measurement tools.

Comparison of HRQOL Between Children With IBD and Healthy Controls

In this subsection, HRQOL in pediatric IBD patients is compared to HRQOL in healthy children.

Five studies that compare HRQOL in children with IBD and healthy children were examined. First, in a study conducted by Marcus et al., 70 children with established IBD are compared to 157 healthy controls based on their responses to the PedsQL 4.0, PedsQL Multidimensional Fatigue Scale, and the Children’s Depression Inventory (short form). Next, Silva et al. assessed QoL in 35 children and adolescents with IBD and compared that to QoL in 62 healthy controls via the PedsQL 4.0 questionnaire as well. Similarly, Loonen et al. recruited 83 children between the ages of 8 and 18 years with IBD in the Netherlands and compared their results on the IMPACT-III questionnaire and on the generic TACQOL to those of a large reference population of 1810 Dutch children. Lucia Casadonte et al. also used the IMPACT-III questionnaire as well as the Children’s Depression Inventory, the PedsQL, and the PedsQL Multidimensional Fatigue to evaluate QoL in 67 children with IBD compared to that of 157 healthy children in Chicago. Lastly, Upton et al. developed a UK-English version of the PedsQL questionnaire and assessed its validity by distributing it to 1034 healthy children as well as 365 children diagnosed with chronic conditions, including 76 with the IBD diagnosis. Each of these studies elaborates on the specific subdomains of HRQOL that are different or the same between children with and without IBD [10-14].

With regard to overall HRQOL scores, children with IBD consistently scored lower than healthy children. Although results varied for each study population, each of the five studies described above reported significantly lower total HRQOL scores than controls on the PedsQL report. Univariate analyses showed that subjects with IBD reported total scores on the PedsQL of 77.79 ±12.34 compared to controls who scored 85.93 ±10.40 (p<0.0006) [14].

Similarly, children with IBD scored significantly lower on the physical health subdomain of the PedsQL in each of the five studies. For example, in the study by Silva et al., children with IBD scored 70.71 ±26.2 vs. 86.33 ±14.1 (p<0.01) in healthy controls, with the lower score indicating worse QoL. Results from the generic TACQOL questionnaire showed lower scores for children with IBD in subdomains of physical health as well. Children with IBD scored statistically significantly lower in both body complaints (22.4 ±6.6 in IBD vs. 24.4±5.1 in controls, p<0.05) and motor functioning (27.9 ±6.4 vs. 30.2 ±2.6, p<0.05) [15]. Overall HRQOL and physical health in particular are thus lower in children with IBD compared to healthy children.

Another important aspect of the PedsQL questionnaire and overall HRQOL is psychosocial health. Overall psychosocial health for children with IBD was found to be consistently lower in each of the five studies. Scores for IBD patients averaged 69.53 ±17.9 compared to 82.88 ±19.4 (p<0.01) in healthy children [12]. Similar statistically significant results were found in the studies by Lucia Casadonte et al. and Upton et al. with regard to overall psychosocial health.

However, some variations exist within the subdomains of psychosocial health. Psychosocial health on the PedsQL entails the mean score of emotional health, social functioning, and school functioning. School functioning includes children’s abilities to keep up with schoolwork as well as the frequency of missing school due to feeling ill or going to the hospital. While patients with IBD scored lower on the subdomain of school functioning across all five studies compared to healthy children, social and emotional health had differing results. Social functioning in the PedsQL examines qualities such as getting along with other children and the ability to make friends. There was no statistically significant difference in social functioning between children with IBD and healthy children [10,11,13].

Lucia Casadonte et al. also found no statistically significant difference in emotional health between the two groups. However, in the studies by Silva, Upton, and Loonen et al., there was a statistically significant difference in emotional health in the PedsQL for children with IBD vs. healthy children. Children scored 68.1 vs. 78.5 respectively (p<0.001) on emotional health [11]. Emotional health on the PedsQL includes feelings of fear and worry about the future. Further, results on the TACQOL showed lower results on negative emotions in general (11.0 ±3.2 vs. 11.9±2.5, p<0.05), but no difference in the domains of cognitive functioning or positive emotions [13]. Thus, while overall psychosocial health and school functioning are consistently lower for children with IBD, emotional and social functioning vary among the studies.

Finally, two of the studies also compared IBD patients with healthy children on the Fatigue Scale. Results from the PedsQL Fatigue scale in both studies showed that IBD patients have lower total scores and subscale scores in general fatigue and sleep/rest fatigue when compared to healthy children (p<0.05). Furthermore, a significant direct relationship was found between increased fatigue and increased prevalence of depression symptoms among IBD patients [12]. Of note, differences in Cognitive Fatigue scores between IBD and control subjects were significant in the Lucia Casadonte study but not significant in the study by Marcus et al. [12,14].

Comparison of HRQOL Between Children With IBD and Those With Other Chronic Conditions

The second goal of this review article is to explore the differences, if any, in HRQOL between pediatric patients who suffer from IBD and other sick children. Here, we discuss a wide range of results obtained from the PedsQL survey across different studies.

Four studies are examined in this subsection. First, a study conducted by Faus et al. in 2014 aimed to compare 60 children with IBD and 60 children who were classified as overweight and obese, based on a BMI greater than the 85th percentile [8]. Second, a cross-sectional study by Marlais et al. compared HRQOL between 40 patients with GERD, 59 with IBD, and 44 with constipation [15]. Finally, two papers published by Youssef et al. were also instrumental in our review. One compared HRQOL between 65 patients with IBD, 65 with FAP, and 56 with GERD [16]. The other explored HRQOL in 42 children with IBD, 80 with chronic constipation, and 56 with GERD [17].

In the domain of physical functioning, obese and IBD patients demonstrated similar scores (79.98 vs. 82.86) [8]. Moreover, comparable scores in physical health were also seen in children with IBD when compared to those with FAP (80.6 vs. 73) [16]. Again, physical health was similar between patients suffering from IBD and those with GERD (82.2 vs. 77.4) [17]. However, a statistically significant difference was observed between patients with IBD and those with chronic constipation (84.6 vs. 75.3, p<0.05) [17]. Physical health is thus comparable between children with IBD and those with other illnesses, such as FAP, GERD, and obesity. However, IBD patients do have better physical health than those with chronic constipation.

Next, there was also no statistically significant difference observed in the category of emotional health across the different studies. For instance, patients with IBD scored 74.58 in the subdomain of emotional functioning, while obese patients scored 71.48 [8]. Patients with IBD and FAP also had comparable scores (78.1 vs. 77.3) in this category [16]. IBD patients scored 78.1, while patients with chronic constipation scored 80.3 in the domain of emotional health [17]. Likewise, patients with IBD and those with GERD also had similar scores (80.3 vs. 71) [17].

Furthermore, similar scores were also demonstrated in the subdomain of social health. IBD patients scored 81.67 in social functioning, while obese children scored 86.47 [8]. Pediatric patients with IBD scored 78.1 and children suffering from FAP scored 77.3 [16]. Patients with IBD and chronic constipation also scored similarly in the category of social health (71.2 vs. 68.4). In addition, IBD pediatric patients scored 89.2, while patients with GERD scored 78.1 in the domain of social functioning. These results were also not statistically significant. In the category of school functioning, comparable scores were also demonstrated between patients with IBD and those with other illnesses. For example, children with IBD had a score of 71.37, while obese patients had a score of 71.78 [8]. Children with IBD scored 73.5 in comparison to 70.8 for children with FAP in the domain of school functioning [16]. In another study, patients suffering from IBD demonstrated a score of 73.5 versus children with constipation who scored 67.8 [17]. Similar scores were also found between patients with IBD (74.7) and those with GERD (67.5) [17].

The total PedsQL score varied among the different patient groups. For example, when comparing children with IBD and those with FAP, patients demonstrated similar total PedsQL scores (83.8 vs. 78.1) [16]. Patients with IBD and obese patients also displayed similar total PedsQL scores (79.3 vs. 76.42) [8]. However, IBD patients demonstrated a greater and statistically significant total PedsQL score when compared to patients with constipation (83.8 vs. 70.4, p<0.05). Furthermore, children with IBD had a greater and statistically significant total PedsQL score when compared with patients suffering from GERD (81.8 vs. 74, p<0.05) [17]. This reveals that patients with IBD have a better HRQOL when compared to children with chronic constipation. Marlais et al. also reported that children with constipation had the lowest quality of life, which is consistent with the findings by Youssef et al. [15].

Moreover, the paper by Faus et al. also stratified their data to identify causes for discrepancies in HRQOL between the patient groups. When the data were stratified for factors related to socioeconomic status, such as race, parent/guardian education, and family income, there was still no statistically significant difference in HRQOL between patients with IBD and obese patients. However, when the data were stratified for gender, statistically significant differences were observed in HRQOL in the domains of physical functioning, emotional functioning, and psychological summary (p<0.05). Females demonstrated lower HRQOL when compared to males in both patient groups with a mean difference of 6.692 (p=0.033) [8].

Discussion

This narrative review examined 10 studies published after 2000 to review HRQOL in pediatric IBD patients as compared to other pediatric patients and healthy children. Initially, we theorized that pediatric patients with IBD have a reduced HRQOL when compared to both healthy and other ill children. Interestingly, our findings showed that while IBD pediatric patients displayed worse HRQOL than healthy children, they showed similar or greater HRQOL when compared with other sick pediatric patients. These results are critical as they can guide intervention in these patients and lead to better outcomes for IBD patients. In this discussion, we aim to explain the key results reported above and their broader impact.

Comparison Among Different Indices

Different surveys and indices were used in the studies to examine HRQOL because there is limited data available evaluating HRQOL in pediatric patients. There are also some advantages in using different indices such as the IMPACT-III, PedsQL survey, and Children’s Depression Inventory, in our review. For instance, it allows us to have a more thorough and comprehensive review as each survey can have a different focus. The IMPACT-III, for instance, is a specialized survey for studying IBD. The Children’s Depression Inventory focuses on the psychosocial health of patients. Finally, the PedsQL survey covers a multitude of subdomains, such as school functioning and emotional health. The PedsQL survey is available in different languages, making it easily accessible. It has also been used to study HRQOL in different pediatric conditions, allowing for more comparisons. Thus, the PedsQL survey can be considered the most advantageous index because it is comprehensive, widespread, and validated. At the same time, a greater push should be made to implement IMPACT-III because it specifically focuses on the effects of IBD.

Factors Associated With Reduced HRQOL in IBD Patients

In our review, we discovered a variety of factors associated with a reduced HRQOL in pediatric patients with IBD. Of note, gender was highlighted as such a factor because females with IBD demonstrated lower HRQOL than their male counterparts [8,10,11,18]. Other studies also noted that HRQOL is worse among females [19]. Reasons for this are multifold; one explanation is that puberty is much more drastic for females than males. The hormonal changes here are also accompanied by more excitability and worsening psychological health [19]. Moreover, females tend to be more concerned with their appearance and well-being [19]. Next, we also found in the literature that coping mechanisms for females involved dealing with issues internally, while males did so externally [19]. Remarkably, all of this contributes to a worsened HRQOL in female patients, when compared to male patients, and thus substantiates our findings.

Another compelling factor associated with a reduced HRQOL that we discovered in our review is fatigue [12,14]. A few different explanations were found for this. Firstly, patients with IBD suffer from worse sleep quality and greater sleep disturbances, which lead to more fatigue as sleep is a predictor of fatigue [20]. Second, patients with lower insulin-like growth factor 1 (IGF-1) levels had significantly greater fatigue. Essentially, this suggests that IGF-1 can influence inflammation and fatigue in these patients [14]. This explanation is especially noteworthy and exciting because it can open the door for more research exploring the biological mechanism behind fatigue and HRQOL. We also found similar findings in the literature as fatigue has been implicated in reduced HRQOL in other chronic conditions, such as cancer, rheumatologic disease, and asthma [12,21,22,23].

Disease activity was also associated with a worse HRQOL in pediatric IBD patients [11,13,24]. Specifically, our review determined that there is an inverse relationship between disease activity and HRQOL. This finding is intuitive as more severe disease corresponds to worse symptoms, complications, and subsequently a reduced HRQOL. On the contrary, we also discovered one finding in the literature that is dissimilar to the ones reported earlier. In the literature, we noted children with IBD scored lower in the school functioning domain when compared to other ill patients [25]. Interestingly, this contrasts with the results we reported as they found that children with IBD had comparable or greater HRQOL than children with other diseases. We also uncovered an explanation for this; impaired school functioning in children with IBD can be due to disease flare-ups, frequent restroom use, and limited participation in gym classes [25].

Factors Associated With Reduced HRQOL in Other Chronically Ill Patients

Notably, we also found that children with IBD had significantly greater HRQOL than children with constipation. This is because of the great stigma associated with constipation [17]. This is consistent with the literature as other patients with constipation have also demonstrated a reduced HRQOL. Chronic constipation is a cumbersome illness and there have even been some suggestions that it can deteriorate HRQOL [26,27,28,29,30]. Additionally, we determined that the stigma around constipation extends deeply as patients are hesitant to discuss their bowel-related symptoms. Instead, they would often choose to self-medicate and postpone meeting with their providers [31]. Interestingly, the symptoms and stigma of constipation can accumulate and consequently worsen HRQOL because patients are more inclined to let their illness worsen than seek a solution. By the same token, we discovered that children with IBD had comparable HRQOL when compared to patients with FAP [16]. Strikingly, our results showed that pediatric patients with FAP have such impaired HRQOL because of their increased sensitivity to pain [32]. This is in line with the findings in the literature; for instance, we determined that these patients have a lower threshold for pain, rectal hypersensitivity, and abnormal instances of referred pain [33,34,35,36]. Markedly, this increased pain sensitivity is due to changes in the central nervous system, increased recruitment of neurons in times of pain, and altered brain-gut communication [37,38,39].

Effect of Age and Adolescence on HRQOL

Remarkably, we observed age and the period of adolescence had inconsistent effects on HRQOL. On the one hand, our findings showed that adolescent IBD patients demonstrated reduced HRQOL when compared to younger children [10]. This is because adolescence is a critical time of development, and these patients are not entirely equipped with dealing with stressors [10]. Thus, the effects of chronic diseases such as IBD are especially great on adolescents and result in reduced HRQOL. Interestingly, this idea is also echoed in the literature. We found that the time of adolescence is associated with hormonal changes that cause physiological discrepancies and imbalances. There is also a gap between their physical and intellectual development, all the while these adolescents are being forced to develop their own values, goals, and identities [19]. Essentially, adolescents are placed in a situation that requires them to grow and mature without having the proper tools. This can exacerbate the stress already associated with a chronic illness and significantly impair HRQOL. On the other hand, we also found that there was no such association between age, adolescence, and reduced HRQOL [40]. This disagreement in the literature should prompt more research into the effects of age and adolescence on HRQOL in IBD patients.

Additional Factors Associated With Reduced HRQOL in IBD Patients

Delving further deep into the literature shows that there are many other factors at play that can explain a decreased HRQOL in pediatric IBD patients when compared to healthy children. For instance, extra-intestinal symptoms, such as those related to the musculoskeletal system, have been correlated with a lower quality of life in IBD patients [41]. We also noted that treatment with steroids is associated with an improved QoL as these patients demonstrated reduced systemic symptoms [18]. Strikingly, the number of flare-ups, recurrences of the disease, perceived stress, and a number of hospitalizations displayed an inverse relationship with HRQOL. On the contrary, the level of education, income, social support, employment, and being male were directly associated with a higher HRQOL [42]. Considerably, psychological variables can also be at play because body appreciation, having a sense of meaning in life, and adopting a positive attitude were associated with a greater HRQOL [43].

Limitations

Our review has a few limitations. These include our selection criteria, which stipulated that only articles that were published in English, after the year 2000, and primary studies were to be included. Our findings may also have been limited by the exclusion of case reports, case series, and studies focused on trials, animal subjects, and medications. Likewise, there were only a limited number of articles available for our review, which meant that there was limited data available to explore. Some of the studies were cross-sectional, which curbed their ability to follow patients up to examine how HRQOL changed over time. Participants in these studies were also chosen from single facilities, which are not representative of the general population. The sample sizes selected were also small and lacked diversity. Some analyses were also hindered by a lack of comparison with healthy children.

## Conclusions

Pediatric IBD is a debilitating and chronic illness. Despite its exhausting clinical course and sequelae, an assessment of QoL in IBD patients is often overlooked. This is one measure that can aid in better gauging a patient’s status and response to interventions. Exacerbating this issue is that HRQOL is a subjective measure, which means it is given less importance by healthcare professionals and researchers. In light of this, this paper aimed to explore and emphasize the significance of HRQOL in these patients. In this review, we found that IBD patients, when compared to healthy children, demonstrated a lower overall QoL, physical health, and psychosocial health. However, IBD patients displayed similar or greater HRQOL scores when compared to other ill children, such as those suffering from FAP, constipation, and GERD. The major factors associated with a decreased HRQOL in IBD patients include age, socioeconomic status, support systems, sex, and associated symptoms, such as fatigue and musculoskeletal symptoms. We also found that emotional and school functioning are two areas that can be improved for children with IBD.

Children should be given the appropriate resources, such as counseling or group therapy, to help better manage their anxieties regarding their health and should be better assisted in school to keep them from falling behind. Going forward, more research should be conducted to gain deeper insights into HRQOL and to further stratify associated risk factors, including socioeconomic status and social indicators. For instance, greater attention should be given to examining musculoskeletal symptoms of IBD as these can impair the physical health aspect of HRQOL. Steroids should be further explored for their role in the treatment of IBD, and not only for use in flare-ups, as they can improve QoL. Furthermore, the scope and utility of HRQOL should be expanded to cover other illnesses, especially in the pediatric community.
